# Accelerated Fatigue Test for Electric Vehicle Reducer Based on the SVR–FDS Method

**DOI:** 10.3390/s24165359

**Published:** 2024-08-19

**Authors:** Yudong Wu, Zhanhao Cui, Wang Yan, Haibo Huang, Weiping Ding

**Affiliations:** 1 School of Intelligent Manufacturing, Chengdu Technological University, Chengdu 611730, China; ydwu@swjtu.edu.cn; 2 School of Mechanical Engineering, Southwest Jiaotong University, Chengdu 610031, China; yw794512@yeah.net (Z.C.); yw1124@swjtu.edu.cn (W.Y.); huanghaibo214@swjtu.edu.cn (H.H.)

**Keywords:** electric vehicle reducer, accelerated fatigue test, SVR–FDS, vibration

## Abstract

The reducer serves as a pivotal component within the power transmission system of electric vehicles. On one hand, it bears the torque load within the power transmission system. On the other hand, it also endures the vibration load transmitted from other vehicle components. Over extended periods, these dynamic loads can cause fatigue damage to the reducer. Therefore, the reliability and durability of the reducer during use are very important for electric vehicles. In order to save time and economic costs, the durability of the reducer is often evaluated through accelerated fatigue testing. However, traditional approaches to accelerated fatigue tests typically only consider the time-domain characteristics of the load, which limits precision and reliability. In this study, an accelerated fatigue test method for electric vehicle reducers based on the SVR–FDS method is proposed to enhance the testing process and ensure the reliability of the results. By utilizing the support vector regression (SVR) model in conjunction with the fatigue damage spectrum (FDS) approach, this method offers a more accurate and efficient way to evaluate the durability of reducers. It has been proved that this method significantly reduces the testing period while maintaining the necessary level of test reliability. The accelerated fatigue test based on the SVR–FDS method represents a valuable approach for assessing the durability of electric vehicle reducers and offering insights into their long-term performance.

## 1. Introduction

As society progresses and product technologies advance, people’s expectations for comfort and reliability in automobiles have also been steadily rising. As a crucial constituent of the electric vehicle’s transmission system, the fatigue life of the reducer significantly impacts the overall performance of the entire transmission system [1,2]. Especially as product quality standards have become increasingly stringent, with the continuous introduction of new laws and regulations in the automotive industry, the reliability and durability of reducers has gained significant attention from manufacturers as well as research and development enterprises [3].

Currently, research on the fatigue characteristics of gearboxes is primarily conducted through simulation calculations and bench testing analyses [4,5,6,7,8]. In simulation calculations, also known as CAE (computer-aided engineering) in engineering, the mechanism of structural damage can be analyzed and clarified. However, model accuracy is greatly affected by the modeling parameters, and some of the parameters are difficult to obtain. As a result, the accuracy of simulation calculations is usually not high enough. A bench test analysis could effectively detect the actual damage to the products during operation. However, the test cycle is long and the test cost is high. Relevant data show that the fatigue life test consumes more than half of the time in the reducer production cycle [9]. Therefore, the design of a speed reducer acceleration fatigue test is of great significance to shorten the production cycle and reduce production cost. Most of the existing accelerated fatigue test methods are designed using the load spectrum editing method or the pseudo-damage value method. The acceleration method based on the pseudo-damage value only considers the time-domain characteristics of the load signal, which leads to a non-negligible error. Additionally, there is a significant difference between the accelerated fatigue life test conditions and the actual working state of the reducer. As a result, the real working conditions of the reducer cannot be accurately simulated. Therefore, it is necessary to explore new methods for accelerated fatigue testing design.

Different from the pseudo-damage value, the fatigue damage spectrum (FDS) is a more accurate method as it considers the frequency domain characteristics of the load signal. The equivalent method, based on the FDS, was first proposed in the French military standards [10], which is used to evaluate the fatigue damage potential of certain components under different dynamic excitations. Since then, the FDS has been applied to the design of durability tests [11]. In the aerospace industry, an accelerated fatigue analysis method based on the FDS has been proposed to evaluate the reliability of electronic control units [12]. The FDS is also used in some test standards, such as MIL-STD-810, which utilizes a simplified formula to calculate the FDS that was proposed by Henderson. Additionally, the Nordic test specifications Aectop-200 (2016) and Aectop-240 (2009) use the damage potential calculation formulas proposed by Lalanne and Henderson, respectively [13].

The FDS provides an effective way to design an acceleration fatigue test for a reducer. However, there remain some challenges in the application of the FDS. The operating conditions of electric vehicles are highly complex, and the frequency domain characteristics of loads vary greatly with different rotational speeds or torques. The FDS of the load on the reducer is a superposition of the FDS under these diverse operating conditions. Consequently, the characteristics of the comprehensive FDS are determined by these varying operating conditions. To make the fatigue damage to the reducer in the acceleration fatigue test more representative of actual operating conditions, it is necessary to obtain the FDS of the reducer under as many different operating conditions as possible. However, conducting tests for all driving conditions would be extremely time-consuming and costly, and it is also impractical to simulate all driving conditions of the vehicles using bench tests. In recent years, data-driven methods have been widely used for extending test data resources or generalizing test data. These methods have been successfully applied in fault diagnosis, sound recognition, and other fields, achieving promising results [14,15]. The data-driven approach provides a potential solution for reconciling the contradiction between test cost and test accuracy in electric vehicle reducer accelerated fatigue tests based on the FDS.

In this study, a new method for electric vehicle reducer accelerated fatigue testing is proposed, which combines the use of the FDS and a data-driven approach. The FDS ensures the accuracy of the accelerated test by capturing the frequency domain characteristics of the different loads. The data-driven method, on the other hand, expands the test data resources based on typical operating conditions, thereby reducing the time and cost required for testing.

The main novel contributions of this paper are as follows:

(1) A new method for the electric vehicle reducer accelerated fatigue test based on the SVR–FDS is proposed. This method can effectively reduce test time consumption while ensuring the accuracy of the accelerated test.
(2) The NEDC (new European driving cycle) test is used to obtain basic data, reflecting the characteristics of electric vehicle operation. The test data is then extended using the SVR method to form a comprehensive database. This database captures the actual working conditions of electric vehicle reducers, and the equivalent FDS established through this approach has significant practical engineering value.
(3) The compressed and equivalent FDS is developed using the multiple linear regression (MLR) method, which enables the design of various acceleration rate test schemes. This approach can meet engineering requirements and allow the design of accelerated fatigue test schemes based on comprehensive consideration of cycle, cost, and fatigue performance.

The remainder of this paper is organized as follows: Section 2 presents the design flow of the accelerated fatigue test method based on the proposed SVR–FDS model. In Section 3, the acquisition of experimental data for the electric vehicle reducer is described, and the test data is extended using the SVR method to form a comprehensive database. Section 4 demonstrates the accelerated fatigue test design process using the MLR method, leveraging the developed database, and the validation of the test scheme. Finally, the paper concludes with a summary in Section 5.

## 2. Methods

**Figure 1:**
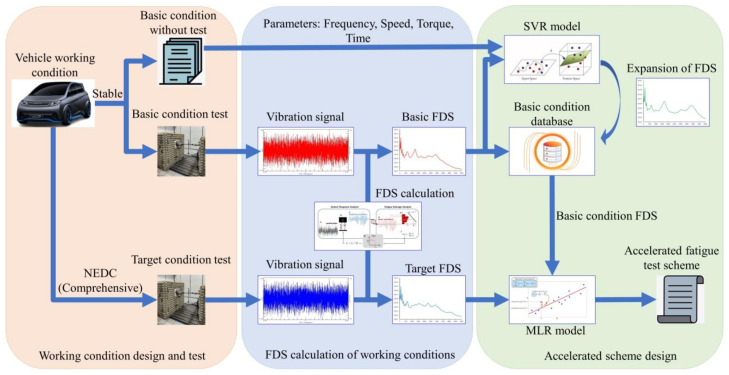
Design flow of the accelerated fatigue test based on the SVR–FDS model.

The accelerated fatigue test design based on the proposed SVR–FDS model can be divided into three steps. Firstly, the working conditions are designed, and the corresponding bench tests are carried out. Secondly, the FDS of the bench test conditions is solved and calculated. Finally, the database is expanded using the SVR model, and the accelerated fatigue test scheme is designed employing the MLR model. The complete design process is illustrated in Figure 1.

The vehicle running conditions required for the design of the accelerated fatigue test are divided into two types. The first type is the target working condition, which can reflect the overall usage of the vehicle. The FDS of the designed accelerated fatigue test should be the same as or similar to the FDS of the target working condition in order to demonstrate that the accelerated fatigue test can reflect the true fatigue characteristics of the reducer during daily driving of the electric vehicle. The second type is the basic working condition under a single speed and torque. To ensure the target damage value is closer to the actual usage situation, the NEDC condition was selected as the target condition to calculate the damage value of the reducer during daily use. It is essential to conduct bench tests to obtain the target working condition and a certain amount of basic working condition data. It should be noted that the severity of the working conditions cannot directly damage the reducer or change its failure mode. The FDS is calculated directly through the vibration acceleration data collected from the reducer housing surface during the bench tests. However, the FDS for the basic working conditions without testing needs to be solved and calculated using the SVR model.

The fatigue damage spectrum (FDS) of the test condition is calculated using the vibration acceleration from the reducer housing surface. The FDS is calculated by determining the number and amplitude of cycles experienced by a virtual series of damped single-degree-of-freedom (SDOF) systems, each tuned to a different natural frequency. This process simulates the response of the device under test (DUT) to the input vibration. The cycles experienced by each SDOF system are then converted to a proportional amount of cumulative damage that would be experienced by the DUT at the corresponding natural frequency. By considering the damage across this range of frequencies, the FDS provides a comprehensive representation of the fatigue damage induced in the DUT due to the input vibration.

Then the stress curve of the system can be calculated by Formula (1) [16]. The rain flow counting method was used to analyze stress distribution on the stress curve [17]. If the S–N curve of the material is assumed to be a straight line in the logarithmic coordinate system, the relationship between stress on the material and the number of cycles is shown in Formula (2). The process of calculating the total damage based on the Miner fatigue damage criterion [18] is shown in Formula (3). Combined with Formulas (1)–(3), the load is applied to a series of SDOF systems with different natural frequencies. The calculation formula of FDS is shown in Formula (4).

(1) σ=k·z

where σ and z are system stress and deformation, respectively, and k is the proportional constant between system stress and deformation.

(2) $$N_{i}\cdot \sigma_{i}^{b}=A^{b}$$

where $\sigma_{i}$ and $N_{i}$ are the stresses and their corresponding number of cycles in the S–N curve, respectively, and A and b are the intercept and slope of the S–N curve, respectively.

**Figure 2:**
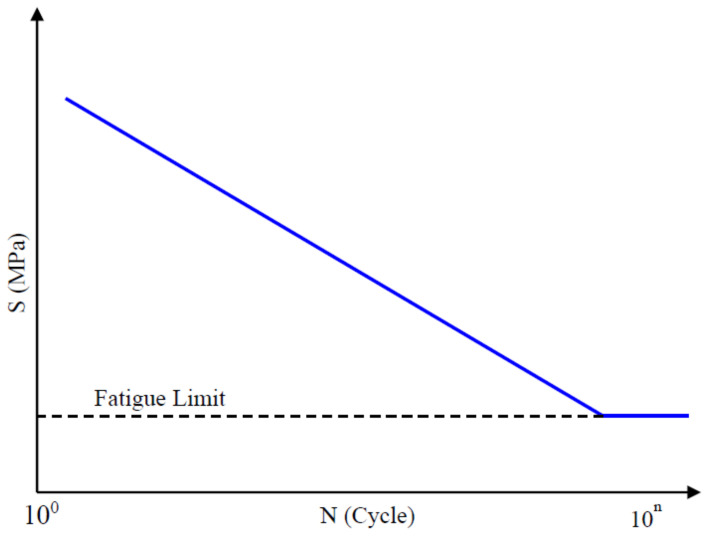
The S–N curve in log-log scale.

The S–N curve is a plot of the magnitude of an alternating stress versus the number of cycles to failure for a given material, shown in Figure 2. Typically, both stress and the number of cycles are displayed on logarithmic scales. Given a load time history and a S–N curve, one can use Miner’s rule to determine the accumulated damage or fatigue life of a mechanical part. The intercept A and slope b of the S–N curve are crucial parameters for characterizing the fatigue performance of materials. The slope of the S–N curve represents the fatigue strength exponent, which indicates the material’s sensitivity to cyclic stress. A higher slope signifies superior fatigue resistance as it means the material can withstand a greater number of cycles before failure at a given stress level. Conversely, a lower slope suggests the material is more prone to fatigue failure. The intercept of the S–N curve denotes the fatigue strength coefficient, which corresponds to the material’s inherent resistance to fatigue at a single cycle. A lower intercept implies the material is more susceptible to fatigue failure at relatively low stress levels. Together, the slope and intercept of the S–N curve provide a comprehensive understanding of a material’s fatigue behavior, enabling engineers to make informed decisions about its suitability for applications subject to cyclic loading.

(3) $$D=\sum_{i}d_{i}=\sum_{i}\frac{n_{i}}{N_{i}}$$

where $n_{i}$ is the cycle number of loads with stress amplitude of $\sigma_{i}$ and $d_{i}$ is the damage caused by loads with stress amplitude of $\sigma_{i}$.

(4) $$FDS\left(f_{n}\right)=\frac{k^{b}}{A^{b}}\sum_{i}n_{i}(f_{n})\cdot z_{i}^{b}(f_{n})$$

where $f_{n}$ is the natural frequency of the SDOF system and $z_{i}$ is the deformation.

The parameters that need to be set in the FDS calculation are the intercept A and slope b of the S–N curve, damping ratio δ, and system force–displacement ratio k.

**Figure 3:**
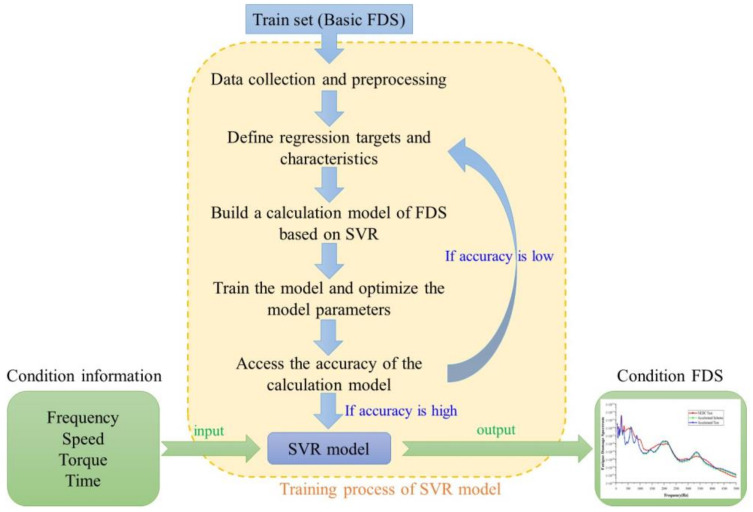
Established SVR model to solve the FDS.

After obtaining the FDS of basic working conditions, the SVR model was established and the FDS of untested working conditions was solved and calculated. The FDS expansion process of working conditions based on the SVR is shown in Figure 3. SVR is a machine learning method that maps the input $x_{i}$ to a high-dimensional feature space through nonlinear mapping [19,20]. After mapping, a linear model is constructed in the high-dimensional feature space. The mapping process is shown in Formula (5).

(5) $$f\left(x\right)=\sum_{i=1}^{d}\omega_{i}\varphi_{i}\left(x\right)+b$$

In Formula (5), d is the dimension of the characteristic space, $\varphi_{i}\left(x\right)$ represents nonlinear mapping, $\omega_{i}$ is the coefficient, and b is the deviation term.

Different from traditional regression models, SVR models can tolerate maximum ε deviation between the regression value $f\left(x\right)$ and the actual value y. The SVR model calculation loss when $\left|f\left(x\right)-y\right|>\varepsilon$ [21,22]. The loss calculation process is shown in Formula (6).

(6) $$L_{\varepsilon }\left(y,f\left(x\right)\right)=\left\{\begin{array}{cc} 0, & \left|y-f\left(x\right)\right|\leq \varepsilon \\ \left|y-f\left(x\right)\right|-\varepsilon , & \mathrm{otherwise} \end{array}\right.$$

In Formula (6), $L_{\varepsilon }$ is the insensitive loss function and ε is the preset threshold. The SVR model used insensitive function in the feature space to make a linear regression and reduce the complexity of the model by minimizing $||\omega |{|}^{2}$ [23,24]. The SVR method can be expressed as solving the optimization problem in the form of Formula (7).

(7) $$\underset{w,b}{min}\frac{1}{2}||\omega |{|}^{2}+C\sum_{i=1}^{m}L_{\varepsilon }\left(y,f\left(x\right)\right)$$

In Formula (7), C is the regularization parameter used to control the compromise between model complexity and approximation error and m is the number of support vectors. Meanwhile, SVR uses the kernel function $K(x_{t},x)$ in the form of Formula (8) to avoid the calculation of mapping function $\varphi_{i}\left(x\right)$ and reduce the computational complexity of high-dimensional hidden space. Commonly used kernel functions are linear kernel, Gaussian kernel, polynomial kernel and so on. Considering that the sample features are few and the nonlinear problem is solved, the Gaussian radial basis function is selected as the kernel function of the SVR model in this paper. In this paper, a Gaussian radial basis function was selected as the kernel function of the SVR model considering that the nonlinear problem is solved and the sample features are few.

(8) $$K\left(x_{t},x\right)=\sum_{i=1}^{d}\varphi_{i}\left(x\right)\varphi_{i}\left(x_{t}\right)$$

The input of the SVR model adopted in this paper included four-dimensional variables; namely, frequency, speed, torque, and time. The output of the model is a one-dimensional target; namely, the FDS under the current working condition. In the process of training the model using basic working condition data, the penalty parameter C and the internal parameter g of the radial basis function are very important to the accuracy of the model. In MATLAB, the best C parameters and g parameters are obtained by cross-validation and grid search. In order to prevent the values that have a great influence on the dependent variable from being shielded, the input and output parameters are normalized in the range of [–1, 1]. The normalization process is shown in Formula (9).

(9) $$x^{*}=2*\frac{x-x_{min}}{x_{max}-x_{min}}-1$$

In Formula (9), $x_{max}$ and $x_{min}$, respectively, represent the maximum and minimum values in the data set, and x and $x^{*},$ respectively, represent the values before and after normalization. If the solution accuracy of the model does not meet the requirements, it is necessary to adjust the SVR model and re-learn the training samples. In this paper, the solution accuracy of the model is evaluated by R-squared ($R^{2}$). The calculation process of $R^{2}$ is shown in Formula (10).

(10) $$R^{2}=1-\frac{\sum (y-\overset{\wedge }{y}{)}^{2}}{\sum (y-\bar{y}{)}^{2}}$$

In Formula (10), y is the actual value, $\bar{y}$ is the mean value of the actual value, and $\overset{\wedge }{y}$ is the solution value.

**Figure 4:**
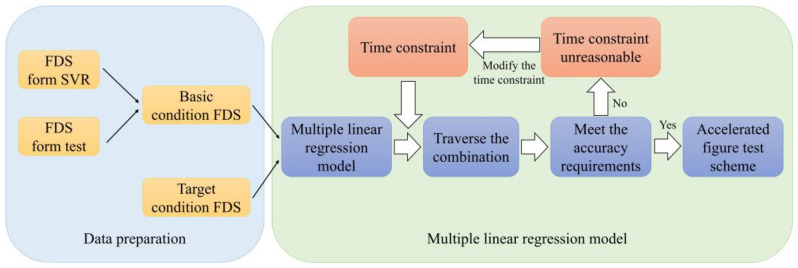
Design test scheme by the MLR model.

After the training of the SVR model, the FDS of untested conditions was solved by the SVR model. The basic condition database for the accelerated fatigue test scheme design was expanded to enrich the characteristic diversity of FDS. Finally, the MLR model was used to fit the target FDS with the basic FDS in the database. The design process of the accelerated fatigue test scheme is shown in Figure 4.

The general form of the MLR equation is shown in Formula (11), in which the parameter $\beta_{j}(j=1,2,\ldots ,k)$ is the partial regression coefficient used to find the solution that minimizes the sum of the remaining squares, as shown in Formula (12). Taking the partial derivative of ${\hat{\beta }}_{1},{\hat{\beta }}_{2},\cdot \cdot \cdot {\hat{\beta }}_{k}$ in the Formula (12) and setting it equal to zero, k equations in the form of Formula (13) can be obtained [25,26,27]. ${\hat{\beta }}_{1},{\hat{\beta }}_{2},\cdot \cdot \cdot {\hat{\beta }}_{k}$ are given by solving this system of equations. The regression results are the running time corresponding to each base condition in the accelerated fatigue test scheme.

(11) $$y_{i}=\beta_{1}+\beta_{2}X_{2i}+\beta_{3}X_{3i}+\cdots +\beta_{k}X_{ki}+\mu_{i}$$

(12) $$min\sum e_{i}^{2}=\sum {\left(Y_{i}-\hat{Y_{i}}\right)}^{2}=\sum {\left[Y_{i}-({\hat{\beta }}_{1}+{\hat{\beta }}_{2}X_{2i}+\cdot \cdot \cdot +{\hat{\beta }}_{k}X_{ki})\right]}^{2}$$

(13) $$\frac{\partial (\sum e_{i}^{2})}{\partial {\hat{\beta }}_{k}}=-2\sum \left(Y_{i}-\hat{Y_{i}}\right)=0$$

The MLR model used time as the boundary condition and fitting accuracy as the constraint condition to solve for the optimal test scheme. The time boundary conditions were set based on engineering requirements. For example, the accelerated fatigue test cycle may be required to be 1/10 or 1/20 of the actual working cycle, corresponding to an acceleration rate of 10-fold and 20-fold, respectively. Within the time requirements set by the engineering specifications, the accelerated fatigue test should be designed to ensure that the FDS of the accelerated fatigue test meets the accuracy requirements. The solution was then evaluated by the coefficient of determination, $R^{2}$, to assess the goodness of fit. The accuracy requirement was that the coefficient of determination was not less than 0.9. If the results do not meet the required accuracy, the time conditions must be reset and the regression calculation repeated. By using different time boundaries, test schemes with varying acceleration rates can be obtained, allowing engineers to select the one that best meets engineering requirements. The accelerated fatigue test scheme can then be designed based on the comprehensive considerations of cycle time, cost, and fatigue performance. This iterative process enables the development of an optimal accelerated test protocol that accurately predicts the product’s fatigue life while balancing practical constraints, such as test duration and budget.

## 3. Database Establishment

### 3.1. Bench Test

**Figure 5:**
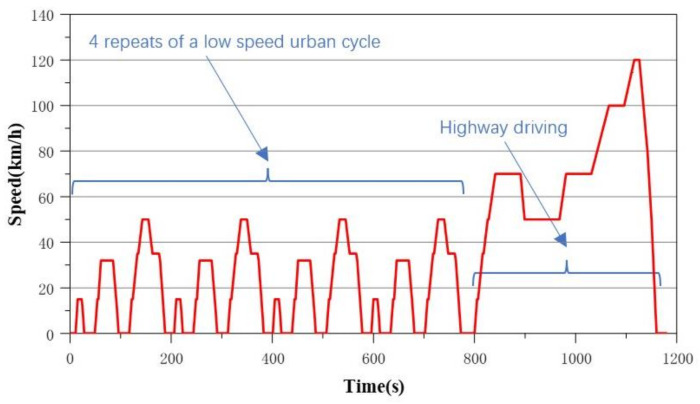
Time–velocity curve of the NEDC.

First and foremost, the damage value of the electric vehicle reducer during operating conditions needs to be obtained. The NEDC condition is set as the bench operating condition to test and calculate the damage value of the reducer during daily use [23,24,25,26,27,28,29,30]. The mean load effect plays an important role in fatigue life predictions as its influence significantly changes high-cycle fatigue behaviors [31,32]. The time–velocity curve of the NEDC cycle is shown in Figure 5. The NEDC condition includes four urban driving cycles and one highway driving cycle (simulation). The mix of the two phases was 66% urban driving and 34% highway driving with a total distance of 11.022 km and a test duration of 19 min and 20 s. The average speed in this cycle was 34 km/h and the maximum speed was 120 km/h.

The input parameters for the bench test were the speed and torque of the electric vehicle reducer. To simulate the vehicle operation on the test bench, the speed and torque of the reducer under different working conditions needed to be calculated according to the vehicle parameters and the reducer parameters. The parameters of the electric vehicle and its reducer are provided in Table 1.

When designing the basic working condition test, the basic working conditions needed to cover the daily operating conditions as comprehensively as possible. However, the severity of the working conditions must not have caused direct damage to the reducer or altered the failure mode of the reducer. A total of 70 groups of basic working conditions were designed, with the stipulation that the input speed, torque, and power do not exceed the rated values. The test table of these basic working conditions is presented in Table 2.

The bench test was conducted in a semi-anechoic chamber, and the driving bench was a three-motor test bench. Triaxial piezoelectric accelerometers were installed at three locations on the reducer housing: the input shaft end, the intermediate shaft end, and the output shaft end. Vibrations in all three directions would have impacted the fatigue of the reducer. These multi-directional vibrations can be modeled as the superposition of three single-degree-of-freedom (SDOF) systems. This approach provides a more authentic representation of the actual loads experienced by the reducer. By capturing the combined effects of vibrations in all three orthogonal axes, the analysis could better reflect the real-world operating conditions and their impact on the reducer’s fatigue life.

**Figure 6:**
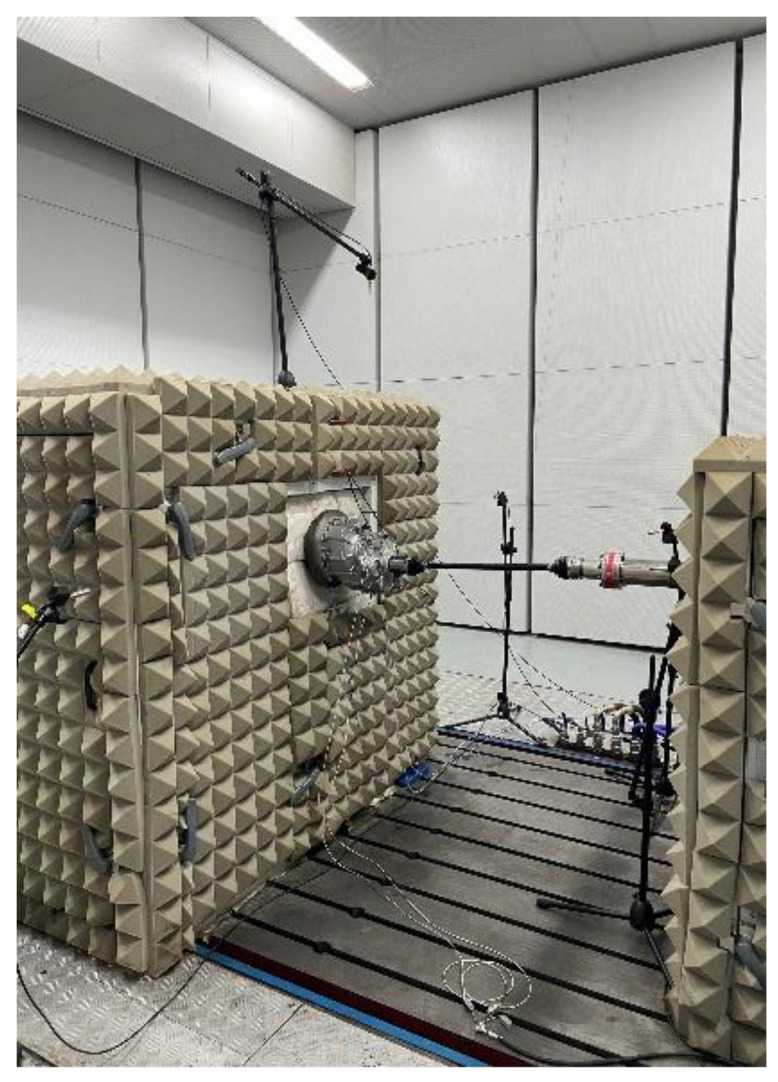
Test bench.

**Figure 7:**
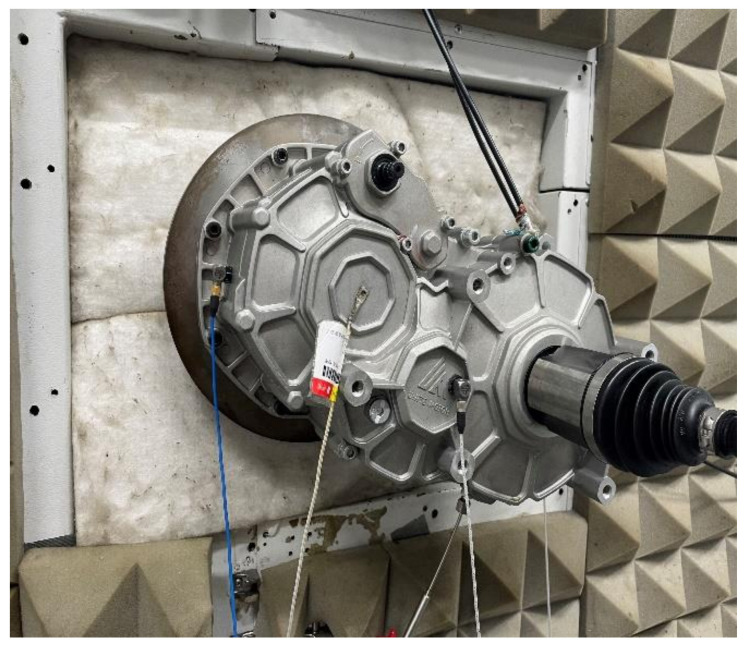
Sensor positions.

The sensor sampling frequency was 20,000 Hz, and the test data acquisition equipment used was the Simcenter SCADAS Mobile. The sampling time for the NEDC condition was 19 min and 20 s while, for the basic working conditions, it was 2 min. The test bench setup and sensor locations are shown in Figure 6 and Figure 7. The sensor information is provided in Table 3. The bench test was conducted for each working condition, and the vibration signal from the intermediate shaft end of the reducer housing was used to calculate the potential damage to the reducer under each condition.

### 3.2. Database Expansion

**Figure 8:**
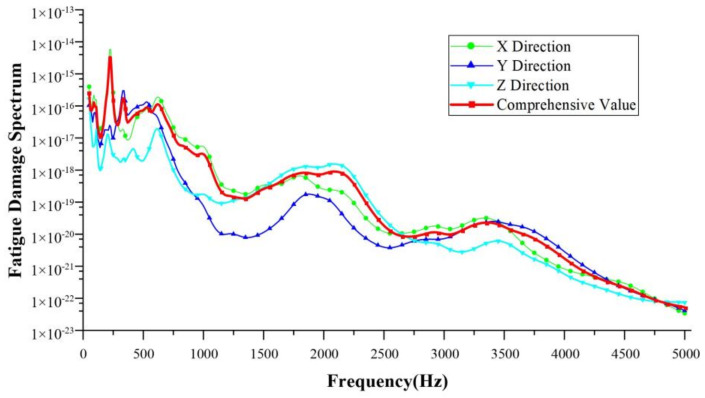
The FDS under the NEDC condition.

**Figure 9:**
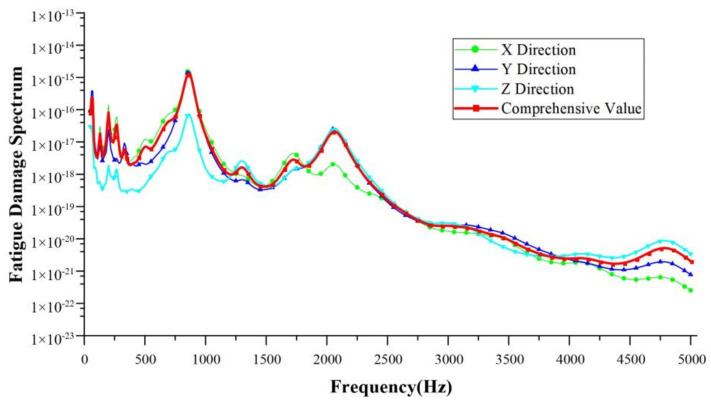
The FDS under basic conditions (4000 rpm–120 Nm) within 10 s.

After collecting the vibration signals from the reducer housing, the FDS of the load on the reducer was calculated for the various working conditions. The frequency analysis range was within 5000 Hz, damping ratio δ = 0.05, proportional coefficient k = 1, S–N curve intercept A = 1, and S–N curve slope b = −4. There was a correlation between the FDS and all three directions. In order to simplify the model, the FDS curves in the three directions were superimposed with vectors to calculate their comprehensive amplitude. The FDS under the NEDC condition is shown in Figure 8. The FDS under basic working condition, with an input speed of 4000 rpm and input torque of 120 Nm within 10 s, is shown in Figure 9.

The SVR model was trained with FDS data obtained from basic working conditions (training dataset) and validated by the data of other basic working conditions (test dataset). The training dataset covers the allowable speed and torque ranges of the reducer, enabling the SVR model to learn as much as possible about the fatigue load characteristics of the reducer under various working conditions, thereby enhancing the generalization capability of the SVR model. The test dataset also covered a variety of working conditions within the speed and torque ranges, which was used to verify that the SVR model could achieve satisfactory accuracy for other different conditions as well. The internal parameters of the SVR model were C = 181 and g = 0.125. The solution model was used to expand the basic working condition database. The expanded working condition table is shown in Table 4. A total of 75 groups of basic conditions were expanded.

**Figure 10:**
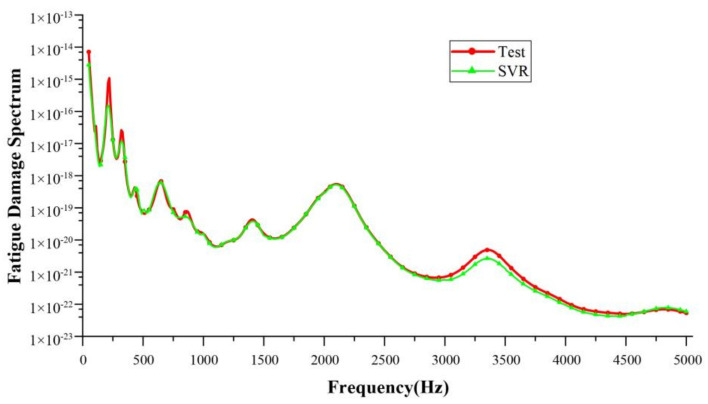
Comparison between the data obtained through SVR and the actual test data.

The accuracy of the SVR model was verified to ensure the reliability of the extended working conditions. In the training of the SVR model, the 6500 rpm–60 Nm working condition was used as the test set data and was not included in the training set. After the training, the solution results of the SVR model were compared with the test results, as shown in Figure 10. The coefficient of determination $R^{2}$ of the solution result of the SVR model was 0.994. The working condition data obtained through the SVR model was found to meet the accuracy requirements.

## 4. Results and Discussion

Each reducer manufacturer has different cost and time requirements for their needs. In an actual accelerated fatigue test scheme, different accelerated fatigue test schemes can be designed for selection. In the previous database established, the target condition FDS and the baseline condition FDS were obtained. Next, the method of MLR was used to design the accelerated fatigue test schemes.

**Figure 11:**
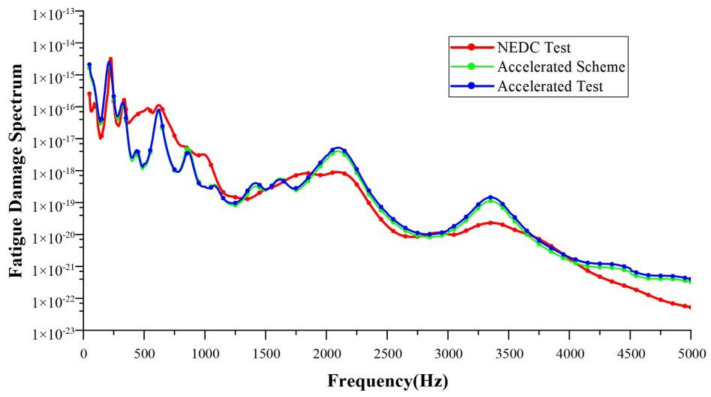
Comparison of FDSs at a 10-fold acceleration rate.

**Figure 12:**
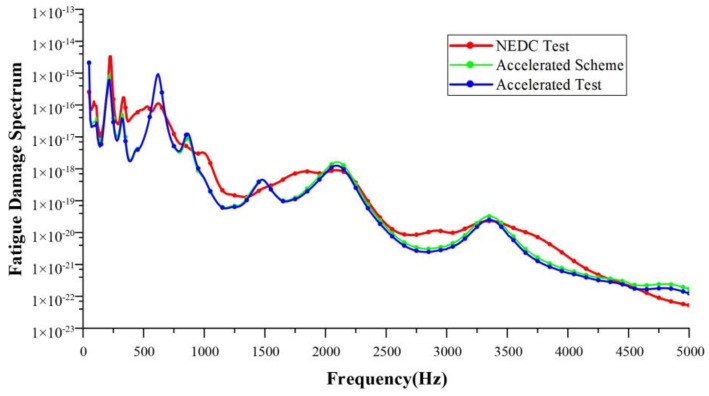
Comparison of FDSs at a 15-fold acceleration rate.

**Figure 13:**
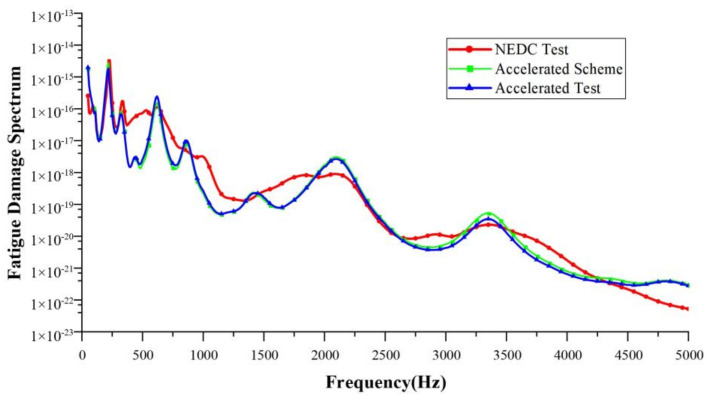
Comparison of FDSs at a 20-fold acceleration rate.

**Figure 14:**
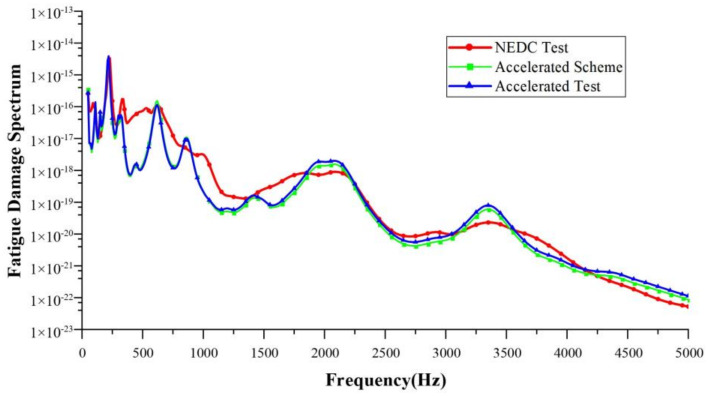
Comparison of FDSs at a 30-fold acceleration rate.

Taking a single NEDC working condition as the target, the target test time was 1160 s. The running time of the corresponding 10-fold acceleration scheme was about 116 s, and that of the 20-fold acceleration scheme was about 58 s. In this paper, the accelerated fatigue test schemes were designed with 10-fold, 15-fold, 20-fold, and 30-fold acceleration rates, respectively. Bench tests were carried out to validate these acceleration schemes. Table 5, Table 6, Table 7 and Table 8 show the test schemes under different acceleration rates. Shown in Figure 11, Figure 12, Figure 13 and Figure 14 are the FDSs obtained from the actual NEDC cycle test (NEDC test, red curve), the simulated FDS of the acceleration test scheme designed by the MLR model (accelerated scheme, green curve), and the actual test FDS of the acceleration test scheme designed using the MLR model (accelerated test, blue curve). We have provided necessary clarification in Section 4.

The coefficient of determination $R^{2}$ is used to evaluate the design accuracy of each acceleration test scheme. Using the FDS obtained from the actual NEDC cycle test as the standard, the $R^{2}$ between the FDS simulated by the acceleration test schemes designed using the MLR method and the standard FDS, as well as the $R^{2}$ between the actual test results of the acceleration test schemes and the standard FDS, were calculated. The results of the coefficient of determination are shown in Table 9. For the acceleration test schemes designed using the MLR method, the $R^{2}$ of both the simulation results and the actual test results are greater than 0.9, which meets engineering requirements.

## 5. Conclusions

(1) This paper proposes an accelerated fatigue testing approach for electric reducers based on the support vector regression (SVR)–fatigue damage spectrum (FDS) method. This method can effectively shorten the fatigue test cycle and reduce the cost of product development and reliability verification.
(2) In this paper, the new European driving cycle (NEDC) condition was proposed as the standard cycle condition for fatigue test analysis of electric vehicle reducers, which aligns with the actual working environment of electric vehicle reducers. The equivalent FDS established by this method has more practical engineering value.
(3) Furthermore, the equivalent FDS is established by the multiple linear regression method, which can be used to design different acceleration rate test schemes. This method can meet the engineering requirements and design the accelerated fatigue test scheme according to the comprehensive requirements of cycle, cost, and fatigue performance.

## Figures and Tables

**Table 1:** Parameters of the electric vehicle.

Parameters	Value
Reducer–transmission ratio i	6.402
Tire model	185/65 R14
Vehicle unloaded mass m (kg)	1090
Transmission efficiency $\eta_{t}$	0.97
Coefficient of rolling resistance f	0.15
Coefficient of air resistance $C_{D}$	0.3
Windward area A (m^2^)	2

**Table 2:** Test list of basic working conditions.

Speed (rpm) /Power (kW)/ Torque (Nm)	20	40	60	80	100	120	140	160	180	200
1000	2.1	4.2	6.3	8.4	10.5	12.6	14.7	16.8	18.9	20.9
2000	4.2	8.4	12.6	16.8	20.9	25.1	29.3	33.5	37.7	41.9
3000	6.3	12.6	18.9	25.1	31.4	37.7	44.0	50.3	56.5	62.8
4000	8.4	16.8	25.1	33.5	41.9	50.3	58.6	/	/	/
5000	10.5	20.9	31.4	41.9	52.4	62.8	/	/	/	/
6000	12.6	25.1	37.7	50.3	62.8	/	/	/	/	/
6500	13.6	27.2	40.8	54.5	/	/	/	/	/	/
7000	14.7	29.3	44.0	58.6	/	/	/	/	/	/
7500	15.7	31.4	47.1	62.8	/	/	/	/	/	/
8000	16.8	33.5	50.3	67.0	/	/	/	/	/	/
8500	17.8	35.6	53.4	/	/	/	/	/	/	/
9000	18.9	37.7	56.5	/	/	/	/	/	/	/

**Table 3:** Sensor information.

Sensor Parameter	Sensor Information
Sensor type	Accelerometer
Model	PCB 356A02
Sensitivity	10 mV/g
Frequency range	1 to 20,000 Hz
Mass loading	10.5 g

**Table 4:** Extended working condition table of the SVR.

Speed (rpm)	Torque (Nm)
5000	25, 30, 35, 45, 50, 55, 65, 70, 75, 85, 90, 95, 105, 110, 115
6000	25, 30, 35, 45, 50, 55, 65, 70, 75, 85, 90, 95, 105, 110
6500	25, 30, 35, 45, 50, 55, 65, 70, 75, 85, 90, 95
7000	25, 30, 35, 45, 55, 65, 75, 85
7500	25, 30, 35, 45, 55, 65, 75
8000	25, 30, 35, 45, 55, 65, 75
8500	25, 30, 35, 45, 55, 65
9000	25, 30, 35, 45, 55, 65

**Table 5:** Accelerated fatigue test scheme at a 10-fold acceleration rate.

Basic Condition	Time
3000 rpm–200 Nm	3.6 s
4000 rpm–140 Nm	2.0 s
5000 rpm–60 Nm	46.9 s
6500 rpm–50 Nm (SVR)	64.9 s

**Table 6:** Accelerated fatigue test scheme at a 15-fold acceleration rate.

Basic Condition	Time
3000 rpm–200 Nm	50.2 s
4000 rpm–140 Nm	3.6 s
6500 rpm–80 Nm	15.3 s
6500 rpm–45 Nm (SVR)	11.7 s

**Table 7:** Accelerated fatigue test scheme at a 20-fold acceleration rate.

Basic Condition	Time
3000 rpm–200 Nm	8.0 s
4000 rpm–140 Nm	3.4 s
6500 rpm–40 Nm	14.7 s
6500 rpm–70 Nm (SVR)	39.2 s

**Table 8:** Accelerated fatigue test scheme at a 30-fold acceleration rate.

Basic Condition	Time
3000 rpm–200 Nm	8.1 s
4000 rpm–140 Nm	4.4 s
6500 rpm–40 Nm	18.8 s
9000 rpm–25 Nm (SVR)	3.6 s

**Table 9:** Calculation results of $R^{2}$.

Acceleration Rate	$\mathrm{Acceleration}\;\mathrm{Scheme}\;R^{2}$	$\mathrm{Verification}\;\mathrm{Test}\;R^{2}$
10-fold	0.917	0.905
15-fold	0.938	0.928
20-fold	0.918	0.921
30-fold	0.919	0.920

## Data Availability

The data used to support the findings of this study are available from the corresponding author upon request.
